# Maternal dexamethasone exposure does not affect glucose tolerance but alters renal haemodynamics in F_1_ rats in a sex‐dependent manner

**DOI:** 10.1002/edm2.450

**Published:** 2023-09-18

**Authors:** Slava A. Malatiali, Narayana Kilarkaje, Maie Al‐Bader

**Affiliations:** ^1^ Department of Physiology, College of Medicine Kuwait University Safat Kuwait; ^2^ Department of Anatomy, College of Medicine Kuwait University Safat Kuwait

**Keywords:** diabetes, prenatal programming, renal haemodynamics, sodium‐glucose transporter‐2

## Abstract

**Introduction:**

Prenatal programming with dexamethasone increases the risk of the development of hyperglycaemia and insulin resistance, leading to diabetes in adulthood. Dexamethasone also causes a decline in renal glomerular filtration in the adult offspring. Sodium‐glucose cotransporter‐2 (SGLT2) plays a significant role in regulating blood glucose and renal haemodynamics in diabetic patients. However, the role of SGLT2 in dexamethasone‐induced programming and the putative sex‐dependent effects on the changes named earlier is unknown. Therefore, this study aimed to investigate the impact of maternal dexamethasone treatment on glucose tolerance, insulin sensitivity, renal perfusion and renal function in adult male and female offspring and the possible contribution of SGLT2 to these changes.

**Methods and Results:**

Pregnant Sprague Dawley rats (F_0_) were treated with either vehicle or dexamethasone (0.2 mg/kg ip) from gestation Day 15 to 20. F_1_ males and F_1_ females were randomly selected from each mother at 4 months of age. There was no change in serum Na^+^, Na^+^ excretion rate, glucose tolerance or insulin sensitivity in F_1_ male or female rats. However, dexamethasone caused significant glomerular hypertrophy and decreases in C_Sinistrin_ and C_PAH_ indicating decreased glomerular filtration rate and renal plasma flow, respectively, in dexamethasone‐treated F1 male but not female rats. Dexamethasone did not affect SGLT2 mRNA or protein expression in F_1_ males or females.

**Conclusion:**

We conclude that dexamethasone‐mediated prenatal programming of glomerular volume, renal function and haemodynamics is sex‐dependent, occurring only in adult male offspring.

## Graphical abstract

Maternal dexamethasone treatment caused glomerular hypertrophy and a decline in renal function and effective renal plasma flow in 4‐month‐old male offspring with no changes detected in females of the same age. The expression of sodium‐glucose transporter‐2 in renal membrane fractions was not affected by dexamethasone treatment suggesting that it does not play a role in the altered haemodynamics in male rats.

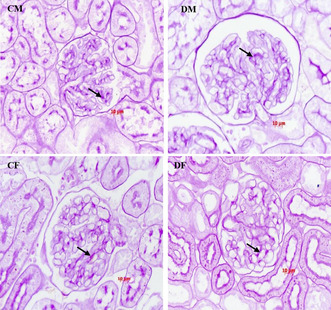

## 1 INTRODUCTION

Pregnant women at risk of premature labour are treated with synthetic glucocorticoids essential for developing many foetal organs.^1^ Synthetic glucocorticoids are not readily metabolized by the placental enzyme‐11β‐hydroxysteroid dehydrogenase‐2^2^; therefore, they may play a role in prenatal programming and have detrimental effects on the foetus, such as intrauterine growth restriction^3^ (IUGR) and the development of diseases later in life such as hypertension and diabetes. Maternal treatment with dexamethasone (Dex) causes an increase in fasting blood glucose level and hyperinsulinaemia in the F_1_ generation, upregulation of hepatic gluconeogenesis enzymes^4^ and a decrease in insulin content in pancreatic β cells.^5^, ^6^

The kidneys could further exacerbate dexamethasone‐induced glucose homeostasis impairment as exposure of cultured cortical tubular cells to dexamethasone causes an increase in glucose synthesis and upregulation of enzymes involved in gluconeogenesis.^7^ Similarly, renal gluconeogenesis increased in diabetic animals and patients (*reviewed in* Sharma and Tiwari 2021^8^). The kidneys can further contribute to hyperglycaemia by altering renal glucose reabsorption. Type 1 diabetic patients showed a higher renal capacity to reabsorb glucose^9^ with increased proximal tubular expression of sodium‐glucose transporter‐2 (SGLT2), responsible for 90% of renal glucose reabsorption, in diabetic animals^10^ and humans.^11^ In addition to altering blood glucose levels, SGLT2 contributes to the hyperfiltration that occurs early in diabetes through tubulogmlomerular feedback.^12^ The increase in proximal Na^+^‐glucose cotransport causes afferent arteriolar dilation leading to an increase in renal blood flow (RBF) and glomerular filtration rate (GFR).^12^ Blocking glucose reabsorption with specific SGLT2 blockers improved glucose tolerance, reduced hyperglycaemia in diabetic rats^13^ and reduced glomerular filtration rate (GFR), albuminuria and renal and glomerular hypertrophy in diabetic Akita mice.^14^ Moreover, with the use of specific SGLT2 blockers, there was a significant decrease in HbA1C and blood glucose levels in Type 1^15^ and Type 2 diabetic patients,^16^, ^17^ with improvement in renal function.^15^, ^18^

The studies mentioned earlier suggest the significance of renal SGLT2 in glucose homeostasis and renal haemodynamics; however, sex differences in the susceptibility to these changes have not been addressed despite the evidence that kidneys of female rats have higher levels of SGLT2 protein expression.^19^ A recent review^20^ concluded that females have higher insulin sensitivity and less susceptibility to diabetes than males. However, the role of sex in the possible development of hyperglycaemia, insulin resistance and diabetes due to prenatal exposure to dexamethasone and the potential contribution of SGLT2 to the above‐mentioned changes are unknown. Therefore, this study aimed to investigate the effect of prenatal dexamethasone treatment on glucose homeostasis, insulin sensitivity, renal haemodynamics and renal expression of SGLT2 in F_1_ adult male and female rats.

## 2 MATERIALS AND METHODS

### 2.1 Animals

Sprague‐Dawley rats were used in this study (200–300 g body weight). The rats were placed in a room with a 12:12 light:dark cycle, kept at 22.3 ± 0.3°C and 31.2 ± 0.8% humidity. The rats had free access to water and standard chow (801151, Special Diets Services). All rats were cared for in accordance with the *Guide for the Care and Use of Laboratory Animals* and all experimental protocols used in this study were approved by the Animal Ethics Committee at Kuwait University, Faculty of Medicine.

For mating, male and female rats were placed in cages at a ratio of 1:3, respectively. The next day, mating was confirmed by the presence of sperm in the vaginal smear and considered Day 0 of pregnancy. The pregnant rats (F_0_) were housed individually in cages until the beginning of the experiment.

### 2.2 Experimental groups

**FIGURE 1:**
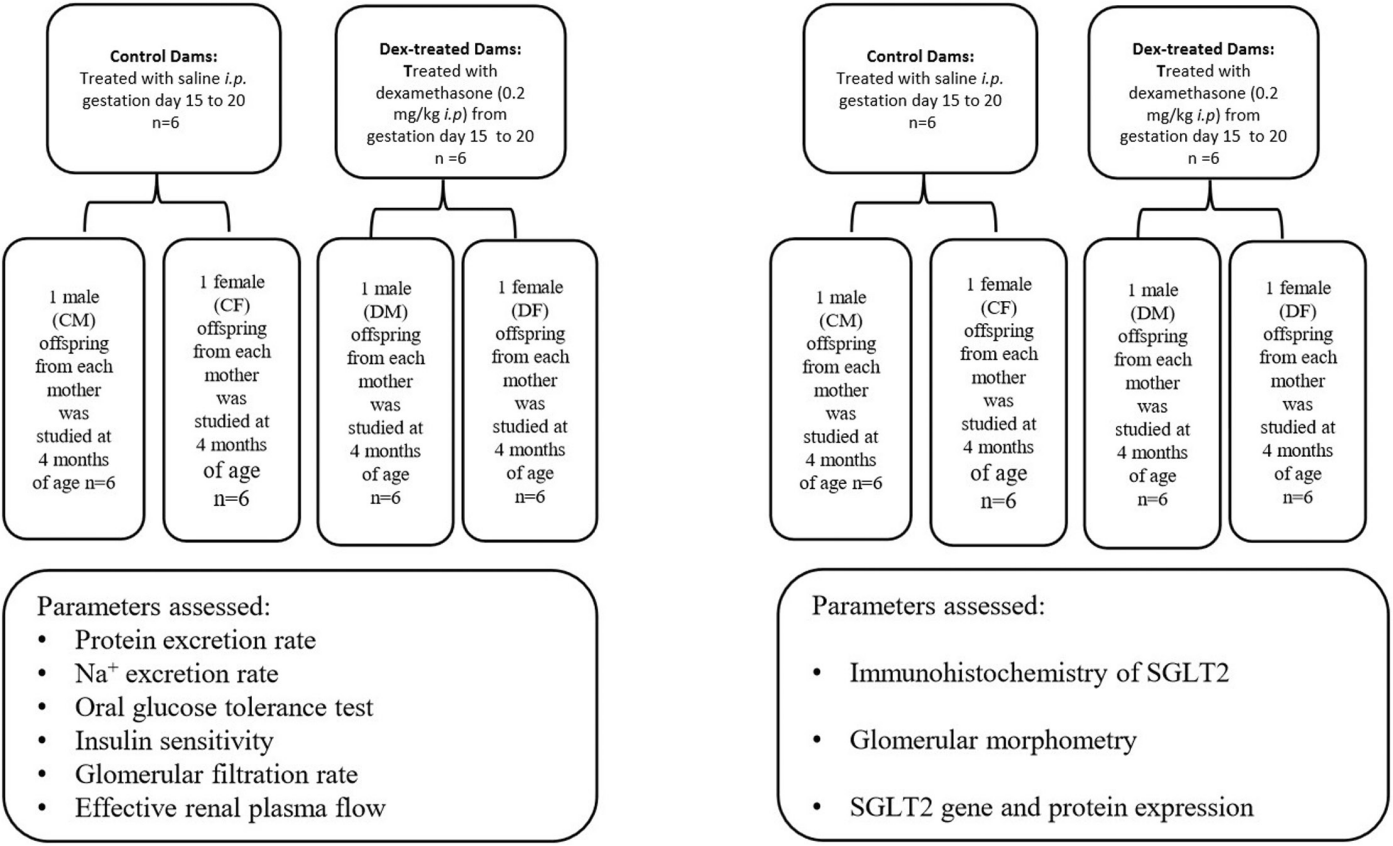
Experimental design. CF, control female; CM, control male; DF, dexamethasone‐treated female; DM, dexamethasone‐treated male.

Pregnant dams were divided into two groups—a control group (C, *n* = 12) treated with vehicle (saline) and another group treated with dexamethasone (Dex, *n* = 12). Dexamethasone 21‐phosphate disodium salt (Cat # D 1159, Sigma‐Aldrich) was administered daily at a dose of 0.2 mg/kg ip^21^ from Day 15 of gestation (dg) to 20dg. The offspring (F_1_ rats) were maintained for four months and randomly divided into two groups—Group 1 contained 12 dams which were further divided into two subgroups—a control group and a Dex‐treated group (*n* = 6/group) to study changes in glucose homeostasis, insulin sensitivity, renal function and renal plasma flow (one F_1_ male and one F_1_ female were randomly chosen from each mother). The Group 2 also contained 12 dams which were further subdivided into two groups as described above (a control and a Dex‐treated group; *n* = 6/group), but their offspring were used to study glomerular morphometric changes, and gene and protein expressions of SGLT2. The experimental design is shown in Figure 1.

### 2.3 Data collection

#### 2.3.1 Body weights, kidney weights and sample collection

Body weights of 4‐month‐old F_1_ males and females (*n* = 12–15) were measured. The F_1_ rats were then euthanised; the right kidney was removed, weighed, and stored at −70°C. The midsection of the left kidney was cut and fixed in 4% paraformaldehyde and processed for paraffin embedding for morphometric measurements and immunohistochemistry. Twenty‐four hours before sample collection, the rats were kept in metabolic cages and 24‐h urine sample was collected.

### 2.4 Glucose homeostasis

#### 2.4.1 Fasting blood glucose and insulin levels

Fasting blood glucose (FBG, G_0_) concentration was measured in 4‐month‐old F_1_ rats in samples taken from the tail vein using a glucometer (GLUCOTREND 2, Roche, Germany). Fasting insulin levels (I_0_) were measured in control and dexamethasone‐treated rats using a rat insulin ELISA Kit (ERINS, Invitrogen). Insulin sensitivity was derived from (G_0_) and (I_0_) levels using quantitative insulin sensitivity check index (QUICKI) = 1/[log(I_0_) + log(G_0_), which is highly correlated with glucose clamp technique.^22^

#### 2.4.2 Oral glucose tolerance test (OGTT)

After 12–14 h of fasting, the blood glucose level was measured (G_0_). Then, a 20% glucose solution at a dose of 2 g/kg body weight was administered to the rats via a gastric tube. Blood glucose level was measured at 15, 30, 60 and 120 min and blood samples were collected, centrifuged and the serum samples were stored at −20°C. The glucose values were plotted against time and the area under the curve (AUC) was calculated as a measure of glucose tolerance as AUC = [G_0_+ (G_30_ x2) + (G_60_x3) + (G_120_ x 4)]/4.^23^

### 2.5 Assessment of renal function.

#### 2.5.1 Protein excretion rate

The rats were placed in metabolic cages and 24‐hour urine was collected. Urinary protein concentration was measured using a Bradford Coomassie blue assay^24^ using a commercially available assay kit (Cat # 23236, Thermo Scientific). Briefly, 300 μL of the Coomassie Plus reagent was added to 10 μL of standard or sample onto the microplate wells. Standards and samples were prepared in duplicates, and after 10 minutes incubation at room temperature the absorbance was measured at 595 nm with Versa Max microplate reader (Molecular Devices, USA). Protein excretion rate (PER) was calculated as: PER = U_p_·V, where U_p_ is urinary protein concentration (mg/ml) and V is urine flow rate (mL/24 h).

#### 2.5.2 Assessment of Na^+^ concentration in serum and urine

Concentration of Na^+^ in serum and 24 h urine samples was measured using an electrolyte measuring system SPOTCHEM ™ EL (SE‐1520, Arkray, Inc). The principle of the method is to convert an electrical potential into an ion concentration using an ion‐selective membrane electrode (SPOTCHEM‐E plate). Serum samples were undiluted while urine samples were diluted 1:1 in distilled water as recommended by the manufacturer.

#### 2.5.3 Sodium excretion rate

Na^+^ excretion rate (E_Na_^+^) was calculated as E_Na_^+^ = U_Na_^+^·V, where U_Na_^+^ is urinary Na^+^ concentration (mM) and V is the urine flow rate (mL/24 h).

#### 2.5.4 Estimation of glomerular filtration rate (GFR) and effective renal plasma flow (RPF)

The GFR and RPF were estimated in 4‐month‐old F_1_ male and F_1_ female of control and dexamethasone‐treated mothers (F_0_) by measuring the clearances of sinistrin (Inutest®; Fresenius‐Kabi, Linz, Austria) and para‐aminohippuric acid (PAH, A1422, Sigma‐Aldrich, Germany), respectively. Each rat was anaesthetized with inactin (Inactin hydrate, 24899957, Sigma‐Aldrich, Germany) at a dose of 100 mg/kg ip. The left carotid artery was catheterized for continuous monitoring of arterial blood pressure. The jugular vein was catheterized and an infusion with sterile Ringer's solution containing 6% albumin at a rate of 60 μL/min was performed for 45–60 min. The urinary bladder was cannulated for urine collection after which infusion of Ringer's solution containing (36 mg/mL) sinistrin and (9 mg/mL) PAH in 1% BSA was started. Priming doses (160 mg/kg) of sinistrin and (8 mg/Kg) PAH were given intravenously. After a 30‐min equilibration period, four samples of urine were collected at 20‐min intervals. At the mid‐point of each period, an arterial blood sample was collected in dry heparinized tubes and centrifuged at 2000 g for 10 min. Concentrations of sinistrin and PAH in plasma and urine samples were measured using the anthrone method (319899, Sigma‐Aldrich, Germany method^25^) and Bratton and Marshall's protocol,^26^ respectively.

### 2.6 Renal expression of SGLT2.

#### 2.6.1 Renal SGLT2 mRNA expression

The SGLT2 mRNA expression was studied in renal tissue by real‐time quantitative polymerase chain reaction (ReT‐PCR). Total RNA was extracted using TRIzol method. The RNA was treated with DNase and reverse transcribed. Real‐time PCR reaction was carried out on a ReT‐PCR system (Applied Biosystems, model 7500) using the following primer—Slc5a2, (Rn00574917_ m1). The reaction was performed using Solis BioDyne PCR master mix and cycled as follows—1 cycle at 50°C for 2 min, 1 cycle at 95°C for 10 min, 60 cycles of alternate 15 secs at 95°C and 1 min at 60°C. Eukaryotic 18S was used as the housekeeping gene (Cat # 4319413E, Applied Biosystems).

#### 2.6.2 Immunohistochemistry for renal expression of SGLT2

Four‐micrometre thick paraffin sections of kidneys were cut and deparaffinized in xylene and dehydrated in descending alcohol grades. The kidney slides were incubated overnight at 4°C with the SGLT2 primary antibody (A gift from Prof. Hermann Koepsell, University of Würzburg (1:250 dilution) in a humidification chamber. The slides were incubated for 30 min with a biotinylated secondary antibody (anti‐rabbit IgG, 1:200 dilution; Vector Laboratories) for 30 min and washed twice with PBS for 5 min each. After incubation for 30 min with the tertiary antibody (Vectastain Elite ABC kit, PK‐6100, Vector Laboratories) and a PBS wash, diaminobenzidine peroxidase substrate (SK‐4100, Vector Laboratories) was applied, and the slides were observed under a light microscope for optimal staining. The slides were washed in distilled water and immersed in haematoxylin for 3 min, followed by a dip in 1% eosin. The sections were dehydrated through ascending grades of alcohol, xylene and mounted with DPX.

### 2.7 Western blotting for SGLT2 protein expression in renal membrane fractions

Renal protein expression of SGLT2 in membrane fractions was studied by western blotting. The renal tissue was homogenized in buffer (10 mM Tris, 1.5 mM EDTA, 10% v/v Glycerol) with 100 μL of 100 mM phenylmethylsulfonyl fluoride (PMSF) protease inhibitors (Pierce protease inhibitor tablets, Cat # SA 2286912, ThermoFisher Scientific). The tissue was centrifuged for 10 min at 4°C at 6000 g. The supernatant was centrifuged at 4°C at 150,000Xg for an hour and the pellet was resuspended in the homogenisation buffer and used for western blotting.^19^ Forty microgram of protein for each sample was loaded on stain‐free gel and electrophoresis was run at 200 V for 30–40 min (Mini‐protein tgx stain‐free gels (Cat # 4568096, Biochemicals and Radiochemicals (BioRad)). The proteins were then transferred to nitrocellulose membranes (Trans‐blot turbo transfer pack cat no: 1704158, BioRad) at 25 V and 1.0 Amp for 30 min, visualized on BioRad ChemiDoc MP Imaging System to measure total protein loaded. After washing the membranes, rabbit polyclonal anti‐SGLT2 antibody was added (Aviva Systems Biology, #ARP43832_ P050) at 1:500 dilution after which the membranes were blocked using 1x Tris‐buffered saline (TBS) (1% casein blocker cat no: 161078, BioRad). Donkey anti‐rabbit IgG secondary antibody (Horseradish Peroxidase‐Linked, Cytiva Amersham ECL, NA934) was added at a dilution of 1:1000. Negative controls with elimination of the primary antibody were run for every gel. The bands were visualized using chemiluminescence on the Chemidoc Imager, then analysed using imaging using Image Lab software. β‐Actin is not a reliable internal control ^27^ especially for membrane fractions as it is variable within the same group (data not shown). Therefore, the expression of each protein was taken as a ratio of SGLT2 band density to the cumulative densities of all proteins loaded per sample.^28^

### 2.8 Glomerular morphological changes: The effect of maternal dexamethasone treatment on glomerular area, volume and mesangial matrix area

Rats were anaesthetized by urethane at a dose of 1.2 g/kg *i.p*. The left kidney was flushed with normal saline, removed and 3 mm‐thick transverse sections were cut and placed in 10% formalin. Three to four days later, the sections were embedded in wax, cut into 4 μm‐thick sections, and mounted on APES (3‐aminopropyltriethoxysilane)‐coated slides for morphological studies. Periodic Acid‐Schiff (PAS) stain was used and the total glomerular tuft area and mesangial matrix areas in 15–20 glomeruli from the renal cortical zone of each rat were measured as previously described^29^ and were assessed by a blind observer.

### 2.9 Statistical analysis

Data were expressed as mean ± SEM for each group. For body weights, kidney weights and physiological data (FBG, PER, glucose and insulin), comparison between groups was performed using one‐way ANOVA followed by Bonferroni or Games‐Howell post hoc tests dependant on the homogeneity of variances. For GFR, RPF, glomerular morphometry and SGLT protein expression, two‐way ANOVA was performed to assess if sex and/or treatment influenced the parameters tested, followed by Bonferroni or Games‐Howell post hoc test as stated in the figures. For the ReT‐PCR protocol, the expression of SGLT2 was normalised to 18S using the 2^−∆∆Ct^ method.^30^ Two‐way ANOVA was performed to assess the effect of sex and/or treatment on SGLT2 mRNA expression. In all experiments, the statistical significance was considered when the *p* value was less than 0.05.

## 3 RESULTS

### 3.1 The effect of maternal dexamethasone exposure on body weights, kidney weights and protein excretion rates in 4‐month‐old F_1_ male and female rats

Exposure to maternal dexamethasone during gestation caused growth retardation, as dexamethasone‐treated pups had significantly (*p* < 0.01) smaller weights (4.6 ± 0.1 g) than controls (5.6 ± 0.2 g) on 21 dg; however, males and females were pooled together. At 4 months of age there was no effect of dexamethasone on body weights in F_1_ females; however, F_1_ males had smaller body weights (*p* < 0.05) and kidney weights (*p* < 0.01) indicating growth restriction with renal growth matching body growth as the ratio of kidney weight to body weight does not change (Table 1). Dexamethasone had no effect on protein excretion rate in either males or females.

**TABLE 1:** Effects of maternal dexamethasone exposure on body and kidney weights and protein excretion rate in 4‐month‐old F_1_ rats.

Group	Body weight (g)	Kidney weight (g)	(KWT/BWT) × 100	PER (mg/day)
CM (*n* = 12)	491.9 ± 13.4	1.38 ± 0.04	0.28 ± 0.01	16.2 ± 1.7
DM (*n* = 14)	441.4 ± 14.8*	1.16 ± 0.06**	0.25 ± 0.02	16.4 ± 1.6
CF (*n* = 13)	265.7 ± 7.7^##^	0.81 ± 0.04^##^	0.31 ± 0.01	3.2 ± 0.80^###^
DF (*n* = 15)	247.2 ± 6.0^##^	0.78 ± 0.04^##^	0.32 ± 0.01	2.6 ± 0.04^###^

Abbreviations: BWT, body weight; CF, control female; CM, control male; DF, dexamethasone‐treated female; DM, dexamethasone‐treated male; KWT, kidney weight; PER, protein excretion rate.

*Note*: The data are expressed as mean ± SE for each group and analysed using one‐way ANOVA followed by Bonferroni post hoc test or Games‐Howell post hoc test.

*p < 0.05; **p < 0.01 control group versus experimental groups; ^##^ *p* < 0.01, ^###^ *p* < 0.001, females compared to males in the same treatment group.

### 3.2 The effect of maternal treatment with dexamethasone on glucose homeostasis in the 4‐month‐old F_1_ males and females

Fasting blood glucose and fasting insulin levels were not affected by maternal treatment with dexamethasone in either F_1_ males or F_1_ females. In addition, GTT and quantitative insulin sensitivity check index showed no change in insulin sensitivity in any of the groups (Table 2).

**TABLE 2:** The effect of maternal treatment on glucose homeostasis in 4‐month‐old F_1_ males and females.

	G_0_ mM	I_0_ (mg/dL)	FI (μU/mL)	QUICKI	AUC (mmol min/L)
CM	5.1 ± 0.1	90.0 ± 2.1	7.6 ± 0.5	0.4 ± 0.003	406.7 ± 12
DM	5.4 ± 0.3	97.2 ± 5.7	6.3 ± 0.3	0.4 ± 0.004	427.7 ± 27
CF	5.8 ± 0.3	98.9 ± 5.7	7.8 ± 1.3	0.3 ± 0.01	404.9 ± 42
DF	5.6 ± 0.2	99.8 ± 3.5	9.6 ± 1.4	0.3 ± 0.01	472.4 ± 27

Abbreviations: CF, control female; CM, control male; DF, dexamethasone‐treated female; DM, dexamethasone‐treated male; G_0_, fasting blood glucose concentration in plasma; I_0_, fasting insulin in serum. *Note*: Results are expressed as mean ± SE. QUICKI = 1/[log(I_0_) 1 log(G_0_)]) is an index of insulin sensitivity.^22^ AUC = area under the curve is calculated as AUC = [G_0_+ (G_30_ × 2) + (G_60_ × 3) + (G_120_ × 4)]/4.^23^ Data are expressed as mean ± SE. *n* = 6–8 in each group. Two‐way ANOVA showed that none of the parameters studied was affected by treatment and/or sex.

### 3.3 The effect of maternal dexamethasone exposure on serum and urine sodium levels in 4‐month‐old F_1_ males and females

Maternal dexamethasone treatment did not affect Na^+^ concentration in serum or urine nor sodium excretion rate (Table 3).

**TABLE 3:** The effect of maternal dexamethasone treatment on serum and urine sodium in 4‐month‐old F_1_ males and females.

	Serum Na^+^ (mM)	Urine Na^+^ (mM)	Urine volume (ml/day)	Na^+^ excretion rate (mmol/day)
CM	138.8 ± 0.8	179.7 ± 16	13.1 ± 0.8	2.4 ± 0.3
DM	139.6 ± 2.3	161.1 ± 25	15.0 ± 1.2	2.3 ± 0.3
CF	141.2 ± 0.1	178.0 ± 26	12.8 ± 1.9	2.0 ± 0.1
DF	139.7 ± 3.7	154.0 ± 17	15.1 ± 0.2	2.2 ± 0.2

Abbreviations: CF, control female; CM, control male; DF, dexamethasone‐treated female; DM, dexamethasone‐treated male *Note*: Data are expressed as mean ± SE. *n* = 6 in each group. Two‐way‐ANOVA showed no effect of treatment and/or sex on any of the parameters measured. There was no significant difference between the groups in any of the parameters studied when compared using Bonferroni post hoc test.

### 3.4 The effect of maternal treatment with dexamethasone on renal function and effective renal plasma flow in 4‐month‐old F_1_ males and females

**FIGURE 2:**
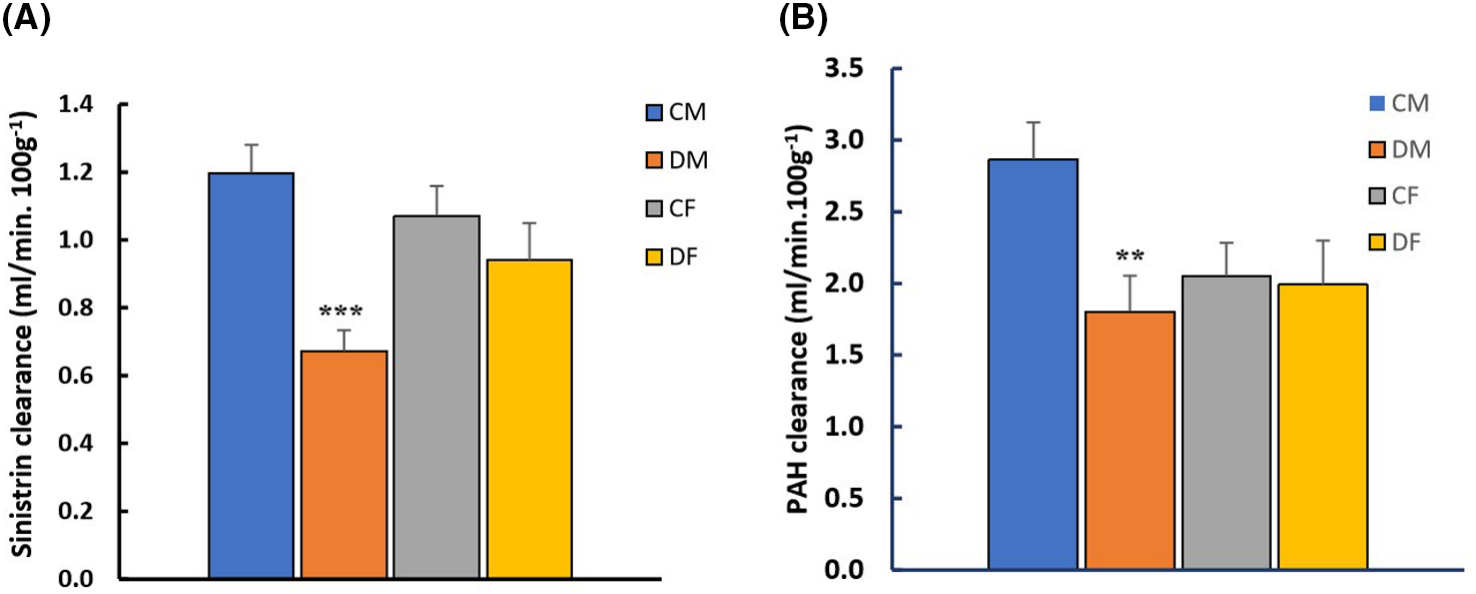
The effect of maternal treatment with dexamethasone on renal plasma clearance of sinistrin (A) and PAH (B) in 4‐month‐old F_1_ males and females. CM, control male; DM, dexamethasone‐treated male; CF, control female; DF, dexamethasone‐treated female. Results are expressed as mean ± SE. *n* = 6 in each group. Data were compared with two‐way ANOVA followed by Bonferroni *post hoc* test and showed that dexamethasone had a significant effect on clearances of sinistrin and PAH while the combination of treatment and sex affects only clearance of sinistrin (GFR). Sex alone has no effect on either parameter. Clearances of sinistrin and PAH significantly decreased only in F_1_ males. **p < 0.01; ***p < 0.001 when compared to controls.

Two‐way ANOVA showed that sex does not affect C_Sinistrin_ or C_PAH,_ however both are affected by Dex treatment. The combination of sex and treatment had an effect on C_Sinistrin_ but not C_PAH_. Maternal dexamethasone treatment caused a significant decrease in C_Sinistrin_ (*p < 0.001)* and C_PAH_ (*p < 0.05)* in 4‐month‐old F_1_ males but not in females, indicating a decline in GFR (Figure 2A) and effective RPF (Figure 2B), respectively.

### 3.5 The effect of maternal treatment with dexamethasone on glomerular morphometry in 4‐month‐old F_1_ males and females.

**FIGURE 3:**
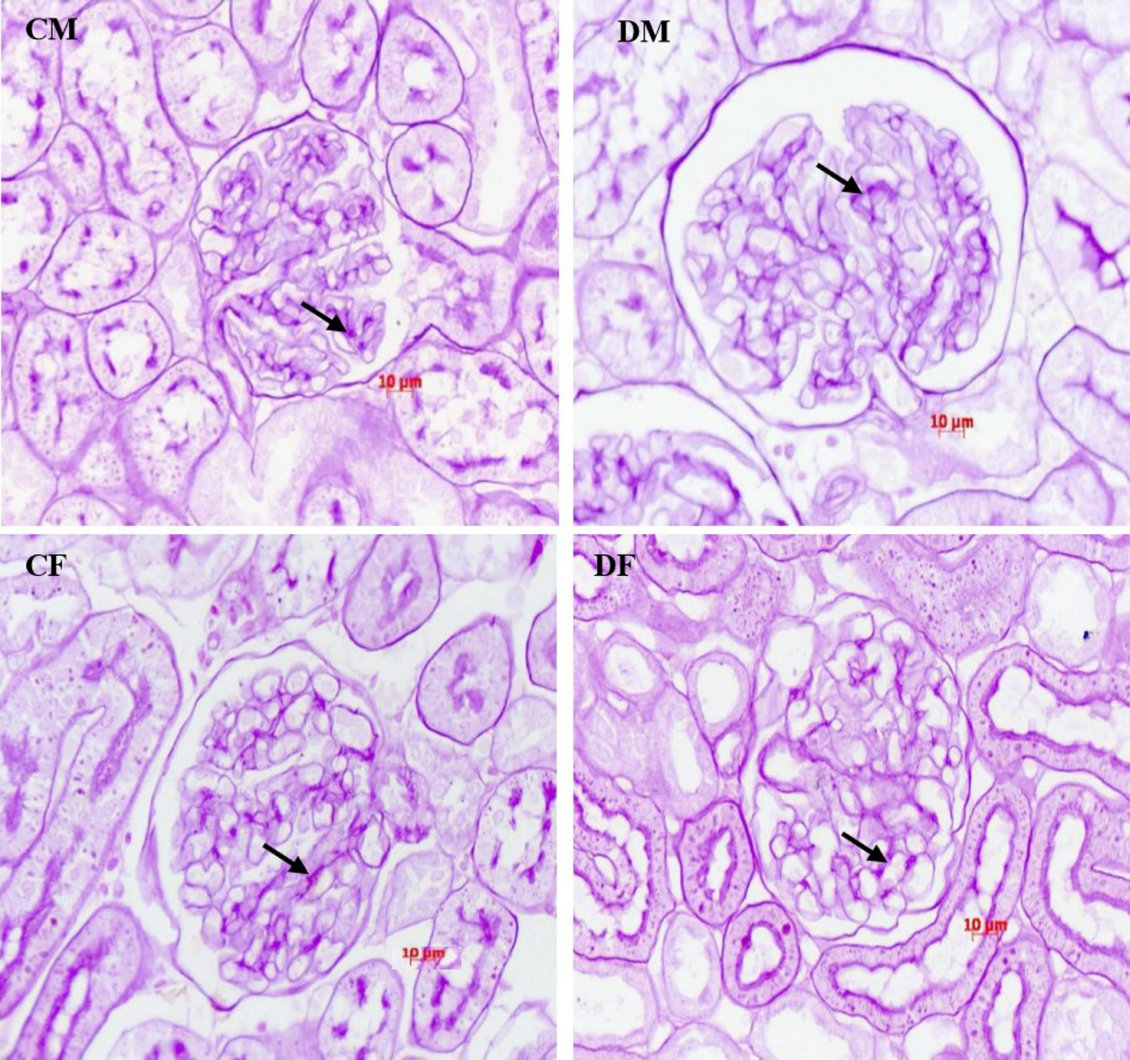
Glomerular morphometric changes in four‐month‐old control males (CM), dexamethasone‐treated male (DM), control female (CF) and dexamethasone‐treated female (DF). There was significant increase in glomerular tuft area (GTA), but no change in and mesangial matrix area (MMA, arrows) or MMA/GTA in dexametbasone‐treated males, No significant changes in glomerular morphometry was observed in 4‐month‐old female rats. (4Km paraffin sections, Periodic‐acid Schiff stain, X 40).

Maternal treatment with dexamethasone caused significant glomerular hypertrophy in F_1_ adult males with substantial increases in the glomerular area (*p* < 0.01) and volume (*p* < 0.01) (Figure 3; Table 4). The treatment did not cause glomerular hypertrophy in F_1_ females, and the mesangial matrix area was not affected by dexamethasone treatment in any group. Two‐way ANOVA showed that the glomerular area and volume are not affected by sex or treatment but rather by the interaction between sex and treatment.

**TABLE 4:** The effect of maternal treatment on glomerular morphometry in 4‐month‐old F_1_ males and females.

	GTA (μm^2^)	MMA (μm^2^)	% MMA/GTA	GV × 10^3^ (μm^3^)
CM (*N* = 96, *n* = 6)	5328.5 ± 234	276.4 ± 16	5.1 ± 0.3	494.2 ± 33
DM (*N* = 147, *n* = 8)	6430.4 ± 217**	329.9 ± 18*	5.1 ± 0.1	656.9 ± 33**
CF (*N* = 96, *n* = 6)	6160.6 ± 221^#^	352.1 ± 25^#^	5.7 ± 0.2	616.6 ± 31^#^
DF (*N* = 81, *n* = 5)	5820 ± 174	353 ± 10^#^	6.1 ± 0.2	553.6 ± 32^#^

Abbreviations: CF, control female; CM, control male; DF, dexamethasone‐treated female; DM, dexamethasone‐treated male; GTA, glomerular tuft area; GV, glomerular volume; MMA, mesangial matrix area.

*Note*: Two‐way ANOVA showed that the interaction between sex and treatment has a significant effect on glomerular area and volume. Results are expressed as mean ± SE and compared using Games‐Howell post hoc test. *p < 0.05, **p < 0.01 when compared to control. #*p* < 0.05 when compared to males in the same treatment group.

### 3.6 The effect of maternal treatment with dexamethasone on SGLT2 gene expression in 4‐month‐old F_1_ males and females

**FIGURE 4:**
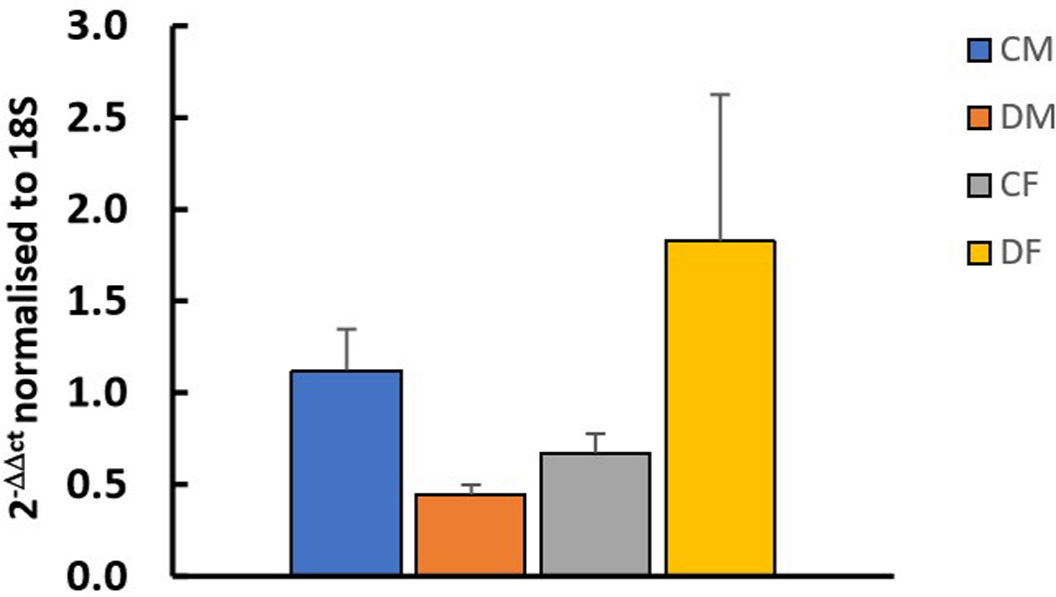
The effect of maternal treatment with dexamethasone on gene expression of SGLT2 in 4‐month‐old F_1_ males and females. CF, control female; CM, control male; DF, dexamethasone‐treated female; DM, dexamethasone‐treated male. *N* = 6 in each group. The results using 2^−ΔΔCt^ method normalised to 18S were compared using 2‐way‐ANOVA analysis of data and showed that there was no effect of sex and/or treatment an SGLT2 mRNA expression.

Analysis of the ReT‐PCR data showed no significant change in SGLT2 mRNA expression in F_1_ males or females exposed to maternal dexamethasone and two‐way ANOVA analysis showed no effect of sex and/or treatment on SGLT2 mRNA expression (Figure 4).

### 3.7 Localisation of SGLT2 protein in F1 males and females

**FIGURE 5:**
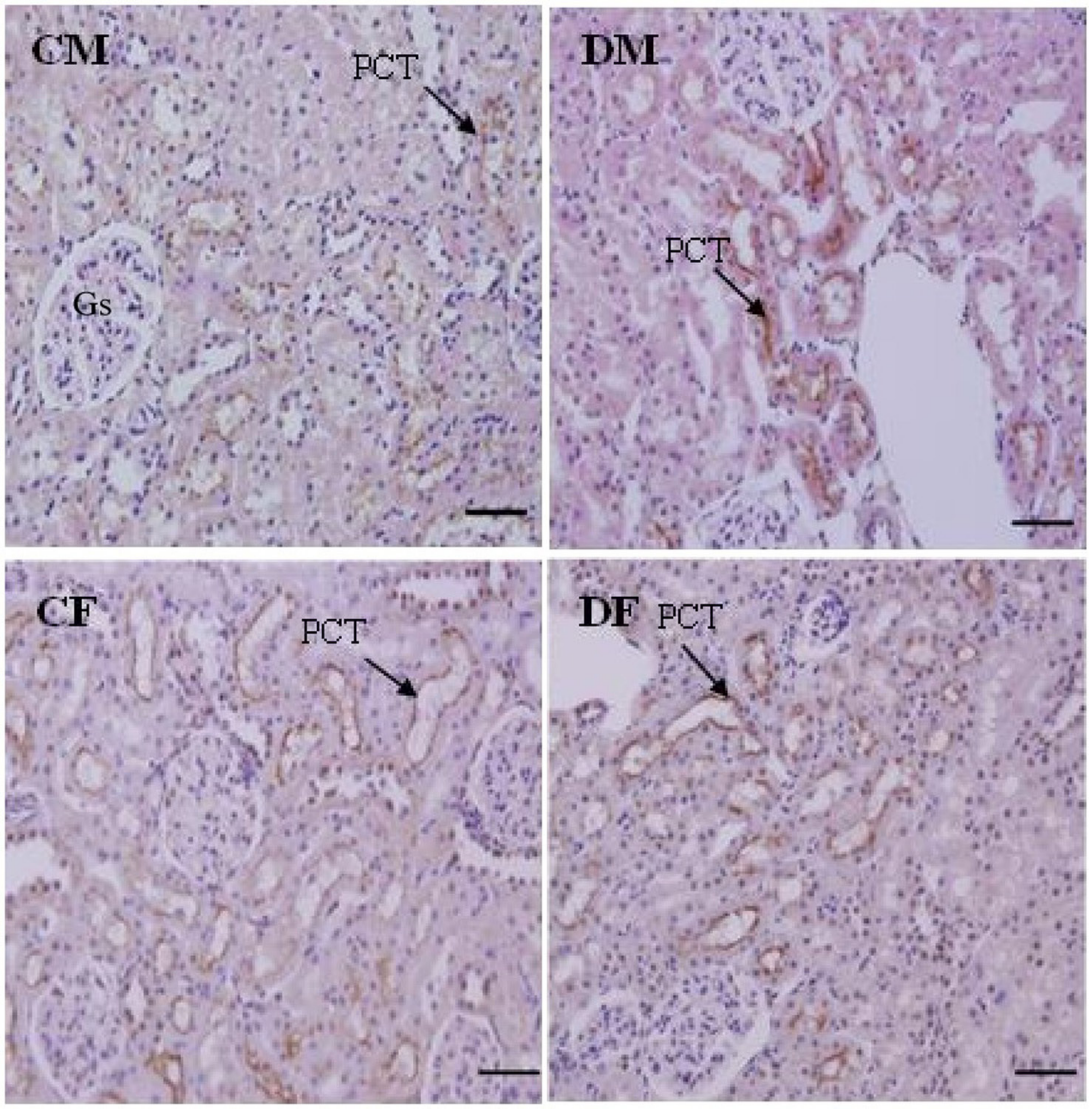
SGLT2 expression in control males (CM), dexamethasone‐treated males (DM), control females (CF) and dexamethasone‐treated females (DF). The protein is expressed (arrows) in SI segments of proximal convoluted tubules (PCT). Note that the glomeruli (Gs) and distal convoluted tubules (DCT) do not express the protein. Counterstained with haematoxylin & eosin, scale bat‐50 nm.

Immunohistochemistry showed that the SGLT2 protein is expressed in S1 segments of proximal convoluted tubules and not expressed in glomeruli (Gs) or distal convoluted tubules (Figure 5).

### 3.8 SGLT2 protein expression in renal membrane fractions in 4‐month‐old F1 males and

**FIGURE 6:**
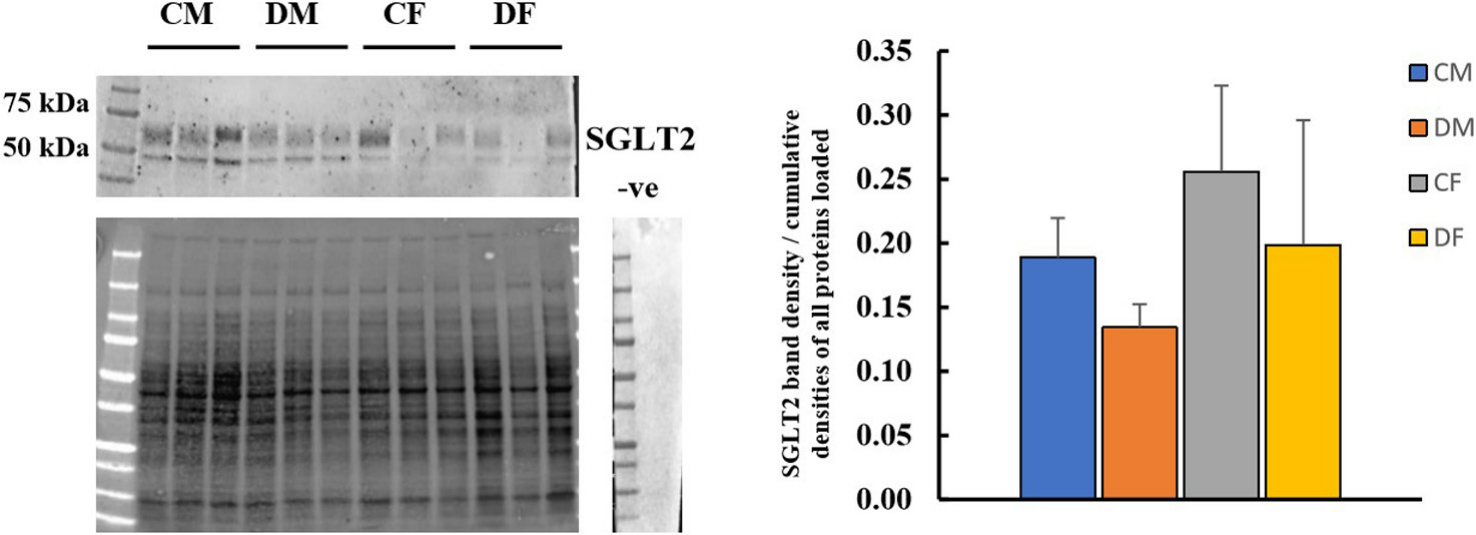
SGLT2 protein expression in renal membrane fractions of control males (CM), dexamethasone‐treated males (DM), control females (CF) and dexamethasone‐treated females (DF). The SGLT2 protein band was at the right size around 66–73 kDa (A). Data (*n* = 6) are expressed as mean ± SEM. SGLT2 expression, taken as the ratio of SGLT2 band density to the cumulative densities of all proteins loaded, was not statistically different in dexamethasone‐treated Fl males or Fl females when compared to their controls (B). Negative controls showed no band at the expected size of SGLT2. Data were compared using two‐way ANOVA and analysis showed that there was no effect of sex and/or treatment on SGLT2 membrane protein expression.

Immunohistochemistry for SGLT2 showed localized expression of SGLT2 in the brush border of the proximal convoluted tubules (Figure 5). Western blotting data indicated no effect of sex and/or treatment on the expression of SGLT2 in renal membrane fractions of 4‐month‐old rats (Figure 6).

## 4 DISCUSSION

This study examined the effect of prenatal dexamethasone treatment on glucose homeostasis and renal function in 4‐month‐old F_1_ males and females. Prenatal programming with glucocorticoids during pregnancy causes IUGR and diseases in the offspring, such as diabetes, that develop later in life.^31^ In our study, at 21 days gestation foetuses of dexamethasone‐exposed mothers were smaller than that of controls, but females and males were studied simultaneously; however, other studies have shown growth retardation in both males and females in the same animal model as ours.^32^ The weights of F_1_ males remained less than that of controls at 4 months of age, similar to earlier reports in which only males had smaller body weights than controls at 6 months.^33^

Maternal treatment with dexamethasone did not affect glucose tolerance or insulin sensitivity either in 4‐month‐old F_1_ males or F_1_ females. In consensus with our results, a previous study showed increased insulin resistance only when dexamethasone treatment was combined with a high‐fat diet in 4‐month‐old male rats.^34^ Conversely, other studies showed increased glucose intolerance in other IUGR models induced either by a low‐protein diet^35^ or intrauterine artery ligation^36^ at 4‐months postnatally; however, these models are incomparable to ours. On the other hand, the same dose of dexamethasone caused hyperglycaemia at six months of age; therefore, this difference may be due to the difference in sample time.^4^

Despite not detecting hyperglycaemia or insulin resistance in our model; the role of prenatal dexamethasone treatment as a risk factor for the development of diabetes is strongly suggested by a random clinical trial on human subjects that showed impaired insulin response to glucose in adults exposed to maternal glucocorticoids.^37^ However, the role of sex in dexamethasone‐induced impaired insulin response was not addressed by that study. Prenatal treatment with dexamethasone did not affect renal handling of proteins as no increase in protein excretion rate was detected at 4 months in either F_1_ males or females. It was noted that PER was significantly higher in males than that in females and is believed to be because of the effect of androgens on the kidneys in males.^38^

In the same model as ours, proteinuria develops only at 6 months of age in male rats.^33^ Similarly, dexamethasone had no effect on sodium levels in serum or sodium excretion rate, a finding that agrees with what was reported earlier.^33^ Maternal dexamethasone treatment did not affect sodium excretion in F_1_ rats. Despite the significant role of SGLT2 in glucose reabsorption, it is responsible for a small portion of proximal tubular sodium reabsorption, therefore any change in SGLT2 activity or expression would have minimal effect on sodium excretion.

Prenatal treatment with dexamethasone caused a significant decrease in GFR in F_1_ males but not in F_1_ females. The studies on the effect of dexamethasone treatment on renal function are contradictory, with some showing a decline in GFR^39^, ^40^ and others not detecting any change in GFR.^21^, ^41^ The differences between these studies and ours are the dose and duration of the dexamethasone treatment. Few studies investigated the role of sex in renal function response to prenatal glucocorticoid treatment programming. Betamethasone caused a reduction in GFR only in adult male sheep.^42^, ^43^ However, in another study, dexamethasone did not affect GFR either in male or female rat offspring, even when combined with a high‐protein diet,^41^ probably due to the lower dose of dexamethasone used (0.1 mg/kg/day) and the shorter duration of the experimental model of 70 days. Recently, in the same model as the one used in this study, dexamethasone caused a decrease in GFR, proteinuria and glomerulosclerosis in male but not female offspring at 1 year postnatally.^40^

Many factors can alter glomerular filtration, such as glomerular surface area, nephron number, renal plasma flow and tubuloglomerular feedback.^12^ The decrease in GFR in dexamethasone‐treated males was attributed to decreased nephron number.^40^ Prenatal dexamethasone treatment caused a decline in nephron number at 70 days^44^ and at 6 months of age in rats,^21^ but in these studies males and females were analysed as one group. Martins et al (2003) showed a decrease in nephron number in 70‐day‐old male but not female offspring when treated with dexamethasone combined with a postnatally high‐protein diet. Similarly, IUGR caused by protein restriction^45^ or intrauterine artery ligation^44^ caused a reduction in nephron number in male but not female offspring, with compensatory glomerular hypertrophy only in male rats.^46^ The effect of dexamethasone on nephron number cannot be verified in our model as it was not measured; however, it may be inferred from the glomerular hypertrophy that occurred only in dexamethasone‐treated males. The decline in renal function cannot be explained solely by the reduction in nephron number as some models reported that GFR remained constant despite lower nephron number.^42^

As for tubular factors, SGLT2 is believed to play a significant role in glucose regulation and renal haemodynamics. Renal SGLT2 expression was not affected by sex in controls, which contradicts what was reported earlier,^19^ probably due to the high variability of SGLT2 expression in females in our model. Dexamethasone treatment had no effect on renal membrane SGLT2 protein expression either, and that was corroborated by the lack of change in renal handling of glucose as it was within normal values in the sera of dexamethasone‐treated F_1_ rats; and the lack of diuresis suggesting normal glucose excretion rate. Therefore, data suggest that SGLT2 does not play a role in the altered renal function seen in male rats.

Our study is the first to show that maternal dexamethasone causes a decrease in effective renal plamsa flow in F1 males, which would be a major cause for the decreased filtration rate in those rats. The decrease in renal plasma flow suggests increased renal vascular resistance although in our study we did not assess vascular resistance. Studies have shown that the direct effect of glucocorticoids on the kidney is a decrease in renal vascular resistance and an increase in renal blood flow,^47^ which is believed to be due to a reduction in the sensitivity to angiotensin II^47^ or increased expression of eNOS.^48^ The only plausible explanation for a decline in renal plasma flow in our study, where dexamethasone caused IUGR, is an increase in preglomerular resistance due to other factors, such as an increase in renal levels of angiotensin II^49^, ^50^ and increased expression of angiotensinogen and angiotensin II type 1 receptor (AT1R),^51^ decrease in renal nitric oxide production,^51^ reduction of vascular compliance,^52^ which is associated with low birth weight^53^ or altered sympathetic activity.^54^

The role of sex in the vascular response to dexamethasone treatment was not addressed by the above‐mentioned studies. Oestrogen and its receptors, which are known to be important regulators of body weight and insulin sensitivity,^55^ were proven to be protective in other models of prenatal programming^56^ and could be the reason behind the sex‐specific renal haemodynamic response to prenatal dexamethasone treatment. Oestradiol activates renal eNOS, which is expressed more in kidneys of female rats^57^ and protects against renal injury.^58^ Few studies explore the possible involvement of RAS in this male‐specific decline in renal function. Ovariectomy increased renal angiotensin converting enzyme (ACE) activity and AT_1_R binding densities and these effects were reversed with oestradiol.^59^ With dexamethasone treatment, female offspring showed lower expression of *Agt* than males^60^ and higher basal expression of angiotensin II type 2 receptor (AT2R) and angiotensin converting enzyme 2, which is the vasodilatory arm of the renin angiotensin system (RAS),^61^ therefore females may respond differently to the activation of RAS.^61^ The role of RAS in the sex specific renal response to maternal dexamethasone treatment should be further investigated.

In conclusion, maternal dexamethasone treatment during late gestation causes glomerular hypertrophy and a decline in renal plasma flow and glomerular filtration rate only in adult male offspring.

## AUTHOR CONTRIBUTIONS

**Slava A Malatiali:** Conceptualization (lead); data curation (lead); formal analysis (lead); funding acquisition (lead); investigation (lead); methodology (lead); project administration (lead); resources (lead); writing – original draft (lead); writing – review and editing (lead). **Narayana Krishna:** Methodology (supporting); writing – review and editing (supporting). **Maie Al‐Bader**: Writing – review and editing (supporting).

## CONFLICT OF INTEREST STATEMENT

The authors declare no conflict of interest.

## Data Availability

The data that support the findings of this study are publicly available at: https://doi.org/10.6084/m9.figshare.22920863.v1.

## References

[1] Rall L , Pictet R , Githens S , Rutter WJ . Glucocorticoids modulate the in vitro development of the embryonic rat pancreas. J Cell Biol. 1977;75(2 Pt 1):398‐409. doi:10.1083/jcb.75.2.398 264117 PMC2109945

[2] Blanford AT , Murphy BE . In vitro metabolism of prednisolone, dexamethasone, betamethasone, and cortisol by the human placenta. Am J Obstet Gynecol. 1977;127(3):264‐267. doi:10.1016/0002-9378(77)90466-5 835623

[3] Reinisch JM , Simon NG , Karow WG , Gandelman R . Prenatal exposure to prednisone in humans and animals retards intrauterine growth. Science. 1978;202(4366):436‐438. doi:10.1126/science.705336 705336

[4] Nyirenda MJ , Lindsay RS , Kenyon CJ , Burchell A , Seckl JR . Glucocorticoid exposure in late gestation permanently programs rat hepatic phosphoenolpyruvate carboxykinase and glucocorticoid receptor expression and causes glucose intolerance in adult offspring. J Clin Invest. 1998;101(10):2174‐2181. doi:10.1172/JCI1567 9593773 PMC508805

[5] Shen CN , Seckl JR , Slack JM , Tosh D . Glucocorticoids suppress beta‐cell development and induce hepatic metaplasia in embryonic pancreas. Biochem J. 2003;375(Pt 1):41‐50. doi:10.1042/bj20030140 14509268 PMC1223676

[6] de Vries A , Holmes MC , Heijnis A , et al. Prenatal dexamethasone exposure induces changes in nonhuman primate offspring cardiometabolic and hypothalamic‐pituitary‐adrenal axis function. J Clin Invest. 2007;117(4):1058‐1067. doi:10.1172/jci30982 17380204 PMC1821070

[7] Kiersztan A , Nagalski A , Nalepa P , et al. DHEA‐induced modulation of renal gluconeogenesis, insulin sensitivity and plasma lipid profile in the control‐ and dexamethasone‐treated rabbits. Metabolic Studies. Biochimie. 2016;121:87‐101. doi:10.1016/j.biochi.2015.11.019 26616007

[8] Sharma R , Sahoo B , Srivastava A , Tiwari S . Reduced insulin signaling and high glucagon in early insulin resistance impaired fast‐fed regulation of renal gluconeogenesis via insulin receptor substrate. J Cell Biochem. 2022;123(8):1327‐1339. doi:10.1002/jcb.30294 35644013

[9] Mogensen CE . Urinary albumin excretion in early and long‐term juvenile diabetes. Scand J Clin Lab Inv. 1971;28(2):182‐193. doi:10.3109/00365517109086899 5130106

[10] Tabatabai NM , Sharma M , Blumenthal SS , Petering DH . Enhanced expressions of sodium‐glucose cotransporters in the kidneys of diabetic Zucker rats. Diabetes Res Clin Pract. 2009;83(1):e27‐e30. doi:10.1016/j.diabres.2008.11.003 19095325 PMC2652566

[11] Rahmoune H , Thompson PW , Ward JM , Smith CD , Hong G , Brown J . Glucose transporters in human renal proximal tubular cells isolated from the urine of patients with non‐insulin‐dependent diabetes. Diabetes. 2005;54(12):3427‐3434. doi:10.2337/diabetes.54.12.3427 16306358

[12] Vallon V , Richter K , Blantz RC , Thomson S , Osswald H . Glomerular hyperfiltration in experimental diabetes mellitus: potential role of tubular reabsorption. J Am Soc Nephrol. 1999;10(12):2569‐2576. doi:10.1681/ASN.V10122569 10589696

[13] Han S , Hagan DL , Taylor JR , et al. Dapagliflozin, a selective SGLT2 inhibitor, improves glucose homeostasis in normal and diabetic rats. Diabetes. 2008;57(6):1723‐1729. doi:10.2337/db07-1472 18356408

[14] Vallon V , Gerasimova M , Rose MA , et al. SGLT2 inhibitor empagliflozin reduces renal growth and albuminuria in proportion to hyperglycemia and prevents glomerular hyperfiltration in diabetic Akita mice. Am J Physiol Renal Physiol. 2014;306(2):F194‐F204. doi:10.1152/ajprenal.00520.2013 24226524 PMC3920018

[15] Cherney DZ , Perkins BA , Soleymanlou N , et al. Renal hemodynamic effect of sodium‐glucose cotransporter 2 inhibition in patients with type 1 diabetes mellitus. Circulation. 2014;129(5):587‐597. doi:10.1161/CIRCULATIONAHA.113.005081 24334175

[16] List JF , Woo V , Morales E , Tang W , Fiedorek FT . Sodium‐glucose cotransport inhibition with dapagliflozin in type 2 diabetes. Diabetes Care. 2009;32(4):650‐657. doi:10.2337/dc08-1863 19114612 PMC2660449

[17] Cefalu WT , Leiter LA , Yoon KH , et al. Efficacy and safety of canagliflozin versus glimepiride in patients with type 2 diabetes inadequately controlled with metformin (CANTATA‐SU): 52 week results from a randomised, double‐blind, phase 3 non‐inferiority trial. Lancet. 2013;382(9896):941‐950. doi:10.1016/S0140-6736(13)60683-2 23850055

[18] Toyama T , Neuen BL , Jun M , et al. Effect of SGLT2 inhibitors on cardiovascular, renal and safety outcomes in patients with type 2 diabetes mellitus and chronic kidney disease: a systematic review and meta‐analysis. Diabetes Obes Metab. 2019;21(5):1237‐1250. doi:10.1111/dom.13648 30697905

[19] Sabolic I , Vrhovac I , Eror DB , et al. Expression of Na^+^‐D‐glucose cotransporter SGLT2 in rodents is kidney‐specific and exhibits sex and species differences. Am J Physiol Cell Physiol. 2012;302(8):C1174‐C1188. doi:10.1152/ajpcell.00450.2011 22262063 PMC3774553

[20] Tramunt B , Smati S , Grandgeorge N , et al. Sex differences in metabolic regulation and diabetes susceptibility. Diabetologia. 2020;63(3):453‐461. doi:10.1007/s00125-019-05040-3 31754750 PMC6997275

[21] Ortiz LA , Quan A , Zarzar F , Weinberg A , Baum M . Prenatal dexamethasone programs hypertension and renal injury in the rat. Hypertension. 2003;41(2):328‐334. doi:10.1161/01.hyp.0000049763.51269.51 12574103 PMC4127977

[22] Katz A , Nambi SS , Mather K , et al. Quantitative insulin sensitivity check index: a simple, accurate method for assessing insulin sensitivity in humans. J Clin Endocrinol Metab. 2000;85(7):2402‐2410. doi:10.1210/jcem.85.7.6661 10902785

[23] Sakaguchi K , Takeda K , Maeda M , et al. Glucose area under the curve during oral glucose tolerance test as an index of glucose intolerance. Diabetol Int. 2016;7(1):53‐58. doi:10.1007/s13340-015-0212-4 30603243 PMC6214468

[24] Bradford MM . A rapid and sensitive method for the quantitation of microgram quantities of protein utilizing the principle of protein‐dye binding. Anal Biochem. 1976;72:248‐254. doi:10.1006/abio.1976.9999 942051

[25] Ludwig TG , Goldberg JV . The anthrone method for the determination of carbohydrates in foods and in oral rinsing. J Dent Res. 1956;35(1):90‐94. doi:10.1177/00220345560350012301 13286391

[26] Bratton AC , Marshall E Jr . A new coupling component for sulfanilamide determination. J Biol Chem. 1939;128(2):537‐550.

[27] Dittmer A , Dittmer J . β‐Actin is not a reliable loading control in Western blot analysis. Electrophoresis. 2006;27(14):2844‐2845. 16688701 10.1002/elps.200500785

[28] Aldridge GM , Podrebarac DM , Greenough WT , Weiler IJ . The use of total protein stains as loading controls: an alternative to high‐abundance single‐protein controls in semi‐quantitative immunoblotting. J Neurosci Methods. 2008;172(2):250‐254. 18571732 10.1016/j.jneumeth.2008.05.00 PMC2567873

[29] Malatiali S , Francis I , Barac‐Nieto M . Phlorizin prevents glomerular hyperfiltration but not hypertrophy in diabetic rats. Exp Diabetes Res. 2008;2008:305403. doi:10.1155/2008/305403 18769499 PMC2522335

[30] Livak KJ , Schmittgen TD . Analysis of relative gene expression data using real‐time quantitative PCR and the 2(‐Delta Delta C(T)) method. Methods. 2001;25(4):402‐408. doi:10.1006/meth.2001.1262 11846609

[31] Martin‐Gronert MS , Ozanne SE . Experimental IUGR and later diabetes. J Intern Med. 2007;261(5):437‐452. doi:10.1111/j.1365-2796.2007.01800.x 17444883

[32] Abul M , Al‐Bader MD , Mouihate A . Prenatal activation of glucocorticoid receptors induces memory impairment in a sex‐dependent manner: role of Cyclooxygenase‐2. Mol Neurobiol. 2022;59(6):3767‐3777. doi:10.1007/s12035-022-02820-8 35396693

[33] Alhamoud I , Legan SK , Gattineni J , Baum M . Sex differences in prenatal programming of hypertension by dexamethasone. Exp Biol Med (Maywood). 2021;246(13):1554‐1562. doi:10.1177/15353702211003294 33794700 PMC8283256

[34] Sheen JM , Hsieh CS , Tain YL , et al. Programming effects of prenatal glucocorticoid exposure with a postnatal high‐fat diet in diabetes mellitus. Int J Mol Sci. 2016;17(4):533. doi:10.3390/ijms17040533 27070590 PMC4848989

[35] Shahkhalili Y , Moulin J , Zbinden I , Aprikian O , Mace K . Comparison of two models of intrauterine growth restriction for early catch‐up growth and later development of glucose intolerance and obesity in rats. Am J Physiol Regul Integr Comp Physiol. 2010;298(1):R141‐R146. doi:10.1152/ajpregu.00128.2009 19889868

[36] Jansson T , Lambert GW . Effect of intrauterine growth restriction on blood pressure, glucose tolerance and sympathetic nervous system activity in the rat at 3‐4 months of age. J Hypertens. 1999;17(9):1239‐1248. doi:10.1097/00004872-199917090-00002 10489100

[37] Dalziel SR , Walker NK , Parag V , et al. Cardiovascular risk factors after antenatal exposure to betamethasone: 30‐year follow‐up of a randomised controlled trial. Lancet. 2005;365(9474):1856‐1862. doi:10.1016/S0140-6736(05)66617-2 15924982

[38] Sellers AL , Goodman HC , Marmorston J , Smith M . Sex difference in proteinuria in the rat. Am J Physiol. 1950;163(3):662‐667. doi:10.1152/ajplegacy.1950.163.3.662 14799645

[39] Celsi G , Kistner A , Aizman R , et al. Prenatal dexamethasone causes oligonephronia, sodium retention, and higher blood pressure in the offspring. Pediatr Res. 1998;44(3):317‐322. doi:10.1203/00006450-199809000-00009 9727707

[40] Jain J , Legan SK , Alhamoud I , Gattineni J , Baum M . Effect of sex on glomerular filtration rate in programmed rats by prenatal dexamethasone. Physiol Rep. 2019;7(12):e14154. doi:10.14814/phy2.14154 31243892 PMC6594923

[41] Martins JP , Monteiro JC , Paixao AD . Renal function in adult rats subjected to prenatal dexamethasone. Clin Exp Pharmacol Physiol Jan‐Feb. 2003;30(1–2):32‐37. doi:10.1046/j.1440-1681.2003.03787.x 12542450

[42] Tang L , Carey LC , Bi J , et al. Gender differences in the effects of antenatal betamethasone exposure on renal function in adult sheep. Am J Physiol Regul Integr Comp Physiol. 2009;296(2):R309‐R317. doi:10.1152/ajpregu.90645.2008 19036827 PMC2643986

[43] Zhang J , Massmann GA , Rose JC , Figueroa JP . Differential effects of clinical doses of antenatal betamethasone on nephron endowment and glomerular filtration rate in adult sheep. Reprod Sci. 2010;17(2):186‐195. doi:10.1177/1933719109351098 19897787 PMC3232065

[44] Ortiz LA , Quan A , Weinberg A , Baum M . Effect of prenatal dexamethasone on rat renal development. Kidney Int. 2001;59(5):1663‐1669. doi:10.1046/j.1523-1755.2001.0590051663.x 11318936 PMC4127466

[45] Black MJ , Lim K , Zimanyi MA , et al. Accelerated age‐related decline in renal and vascular function in female rats following early‐life growth restriction. Am J Physiol Regul Integr Comp Physiol. 2015;309(9):R1153‐R1161. doi:10.1152/ajpregu.00403.2014 26377562

[46] Schreuder MF , Nyengaard JR , Fodor M , van Wijk JA , Delemarre‐van de Waal HA . Glomerular number and function are influenced by spontaneous and induced low birth weight in rats. J Am Soc Nephrol. 2005;16(10):2913‐2919. doi:10.1681/ASN.2004100875 16093454

[47] Kubota E , Hayashi K , Matsuda H , et al. Role of intrarenal angiotensin II in glucocorticoid‐induced renal vasodilation. Clin Exp Nephrol. 2001;5(3):186‐192. doi:10.1007/s101570170009

[48] Bobadilla NA , Tapia E , Jimenez F , et al. Dexamethasone increases eNOS gene expression and prevents renal vasoconstriction induced by cyclosporin. Am J Physiol. 1999;277(3):F464‐F471. doi:10.1152/ajprenal.1999.277.3.F464 10484530

[49] Dagan A , Gattineni J , Habib S , Baum M . Effect of prenatal dexamethasone on postnatal serum and urinary angiotensin II levels. Am J Hypertens. 2010;23(4):420‐424. doi:10.1038/ajh.2009.274 20075846 PMC4130337

[50] South AM , Nixon PA , Chappell MC , et al. Antenatal corticosteroids and the renin‐angiotensin‐aldosterone system in adolescents born preterm. Pediatr Res. 2017;81(1–1):88‐93. doi:10.1038/pr.2016.179 27636897 PMC5646358

[51] Tain YL , Huang LT , Lee CT , Chan JY , Hsu CN . Maternal citrulline supplementation prevents prenatal N(G)‐nitro‐L‐arginine‐methyl ester (L‐NAME)‐induced programmed hypertension in rats. Biol Reprod. 2015;92(1):7. doi:10.1095/biolreprod.114.121384 25395680

[52] O'Sullivan L , Cuffe JS , Paravicini TM , et al. Prenatal exposure to dexamethasone in the mouse alters cardiac growth patterns and increases pulse pressure in aged male offspring. PloS One. 2013;8(7):e69149. doi:10.1371/journal.pone.0069149 23935943 PMC3723833

[53] Mzayek F , Sherwin R , Hughes J , et al. The association of birth weight with arterial stiffness at mid‐adulthood: the Bogalusa heart study. J Epidemiol Community Health. 2009;63(9):729‐733. doi:10.1136/jech.2008.084475 19429574

[54] Dagan A , Kwon HM , Dwarakanath V , Baum M . Effect of renal denervation on prenatal programming of hypertension and renal tubular transporter abundance. Am J Physiol Renal Physiol. 2008;295(1):F29‐F34. doi:10.1152/ajprenal.00123.2008 18400872 PMC4063419

[55] Meyer MR , Clegg DJ , Prossnitz ER , Barton M . Obesity, insulin resistance and diabetes: sex differences and role of oestrogen receptors. Acta Physiol (Oxf). 2011;203(1):259‐269. doi:10.1111/j.1748-1716.2010.02237.x 21281456 PMC3110567

[56] Ojeda NB , Grigore D , Robertson EB , Alexander BT . Estrogen protects against increased blood pressure in postpubertal female growth restricted offspring. Hypertension. 2007;50(4):679‐685. doi:10.1161/HYPERTENSIONAHA.107.091785 17724277 PMC2850594

[57] Reckelhoff JF , Hennington BS , Moore AG , Blanchard EJ , Cameron J . Gender differences in the renal nitric oxide (NO) system: dissociation between expression of endothelial NO synthase and renal hemodynamic response to NO synthase inhibition. Am J Hypertens. 1998;11(1 Pt 1):97‐104. doi:10.1016/s0895-7061(97)00360-9 9504456

[58] Singh AP , Singh N , Pathak D , Bedi PMS . Estradiol attenuates ischemia reperfusion‐induced acute kidney injury through PPAR‐gamma stimulated eNOS activation in rats. Mol Cell Biochem. 2019;453(1–2):1‐9. doi:10.1007/s11010-018-3427-4 30194582

[59] Dean SA , Tan J , O'Brien ER , Leenen FH . 17beta‐estradiol downregulates tissue angiotensin‐converting enzyme and ANG II type 1 receptor in female rats. Am J Physiol Regul Integr Comp Physiol. 2005;288(3):R759‐R766. doi:10.1152/ajpregu.00595.2004 15550614

[60] Tain YL , Wu MS , Lin YJ . Sex differences in renal transcriptome and programmed hypertension in offspring exposed to prenatal dexamethasone. Steroids. 2016;115:40‐46. doi:10.1016/j.steroids.2016.08.006 27521802

[61] Sampson AK , Moritz KM , Jones ES , Flower RL , Widdop RE , Denton KM . Enhanced angiotensin II type 2 receptor mechanisms mediate decreases in arterial pressure attributable to chronic low‐dose angiotensin II in female rats. Hypertension. 2008;52(4):666‐671. doi:10.1161/HYPERTENSIONAHA.108.114058 18711010

